# Prevalence, outcome and conduct of in-hospital cardiopulmonary resuscitation in government hospitals of Nepal

**DOI:** 10.1371/journal.pone.0316950

**Published:** 2025-01-31

**Authors:** Pawan Kumar Hamal, Surendra Kunwar, Kapil Gautam, Ramesh Bhattarai, Rupesh Kumar Yadav, Ritesh Lamsal, Radeep Singh, Sonam Pathak, Nabin Pokhrel

**Affiliations:** 1 National Academy of Medical Sciences, National Trauma Center, Kathmandu, Nepal; 2 Consultant Anesthesiologist, Lumbini Provincial Hospital, Butwal, Nepal; 3 Consultant Anesthesiologist, Seti Provincial Hospital, Dhangadi, Nepal; 4 Consultant Anesthesiologist, Karnali Academy of Health Sciences, Jumla, Nepal; 5 Tribhuvan University Teaching Hospital, Kathmandu, Nepal; 6 Consultant Anesthesiologist, National Trauma Center, Kathmandu, Nepal; 7 University of Potomac, Washington, DC, United States of America; Medical University of Vienna, AUSTRIA

## Abstract

**Introduction:**

Cardiopulmonary resuscitation (CPR) is an evidence-based intervention that saves lives. In low- and middle-income countries like Nepal, the occurrence of the problem and its outcome are seldom studied. The study aims to highlight the prevalence, performance, and outcome of CPR in government hospitals of Nepal.

**Methods:**

A mixed method study was done for two months in central and provincial government hospitals of Nepal. A total of 80 resuscitations were evaluated using a questionnaire based on the American Heart Association 2020 guidelines for cardiopulmonary resuscitation. An in-depth interview was conducted with 15 active participants of the resuscitation in different sites. Thematic analysis was done using the framework of the chain of survival of arrest victims.

**Results:**

The overall prevalence of CPR was found to be 1.92% [95% CI: 0.01,0.02] with 5.4% in central hospitals and 0.65% in provincial hospitals with 60% cardiac arrests occurring in the intensive care unit. Estimated time from recognition of the arrest to initiating CPR was 1.9 ±1.4 minutes. Asystole 66.25% was the commonest arrest rhythm and 21.25% had difficulty interpreting rhythm. Only 11.25% of the victims had return of spontaneous circulation and were subsequently transferred for post-arrest care. The qualitative analysis highlighted the lack of trained staff, a dedicated system, feedback mechanism, and provision of post-arrest care.

**Conclusion:**

Across various level of Nepal’s healthcare system, cardiopulmonary resuscitation is prevalent with poor performance and outcome. To improve outcomes, it is essential to implement standardized procedures and ensure high quality resuscitation delivery before and after the event supported by well-trained healthcare personnel and adequate infrastructure.

## Introduction

Cardiopulmonary resuscitation (CPR) is a cost-effective intervention that saves lives. The reported incidences of In-hospital cardiac arrest (IHCA) vary globally, ranging from 1.2–9 per 1000 admissions [1]. Specifically, the DANARREST (Danish in-hospital cardiac arrest registry) nationwide registry reports prevalence of 1.8 per 1000 admission [2], Japan reports 5.1 per 1000 hospital admission [3], the Get-With-The-Guidelines-Resuscitation (GWTG-R) reports 9.7 per 1000 hospital admissions in USA [4] and UK National Cardiac Arrest Audit (NCAA) reports 1–1.6 per 1000 admissions [5]. In study done in Uganda, IHCA was found to be 2.3% with return of spontaneous circulation (ROSC) in 7.4% cardiac arrest [6]. Arrest Outcome Consortium Registry Analysis (AOCRA) 2022 from India reports 2235 cardiac arrest which includes 1998 IHCA from 2017 to 2022, had 16.7% ROSC and 6.6% having good neurological outcome at discharge [7]. A systematic review which analyses 40 IHCA studies from 1985–2018 reports 13.4% one-year survival with wide variation across regions [8]. Studies based on CPR in Nepal are mostly done in academic institutions mainly on pediatric and neonatal populations, most often highlighting the performance of the trainees, unavailability of equipment, and lack of adherence to standard guidelines [9–11]. There is dearth of literature in low and middle income country particularly Nepal regarding the burden of the problem in and outside the hospital, and even if intervention is done in the hospital setting, standardization and the outcome of the patients are hardly measured. The study aims to highlight the prevalence, performance, and outcome of CPR in government hospitals of Nepal.

## Methods

### Study design

A mixed-method design study was done during the period of 2 months (June 15, 2021- August 15, 2021) prospectively. Quantitative part of the study included an observational study which was done using face to face interview using a questionnaire with one of the participants of the CPR. The ‘participant’ was defined as the healthcare personnel who took active participation during the time of CPR, either as chest compressor, airway manager, drug administrator, defibrillator, data collector or as the head of the resuscitation team. A phenomenological design was used to assess the qualitative part of the study using an in-depth interview of the resuscitation performer.

### Settings

The government hospitals were sampled in two strata—central hospitals under the federal government, and provincial hospitals under the provincial government. Facilities were randomly chosen from the two strata of central and provincial hospitals from the list of hospitals from the website of the Ministry of Health and Population and after confirmation of the presence of critical care facility for post-resuscitation care. For the study purpose, two central hospitals, one inside and other outside the capital city Kathmandu was taken. Two provincial hospitals outside Kathmandu were taken as per the list of Government of Nepal. Two central hospitals of the Government of Nepal, National Academy of Medical Sciences, National Trauma Center, Kathmandu, Nepal (inside the capital) and Karnali Academy of Health Sciences, Jumla, Nepal (outside the capital) were selected randomly for the study. Two provincial hospitals Seti Provincial Hospital, Dhangadi and Lumbini Provincial Hospital, Butwal were selected.

### Sample size and sampling

Registry-based data from the United Kingdom indicate a cardiac arrest prevalence of 1.6 per 1000 population per year [5]. Similarly, in the United States, the estimated annual incidence of cardiac arrest is 9–10 arrests per 1000 admissions. If we look at the prevalence of IHCA in an African hospital study, it is 2.3% [6]. Similarly, in a study done in India, the prevalence of IHCA is as high as 6.7% [12]. So, we took a value of around 5% as the estimated value of prevalence of cardiac arrest in our study. Taking the prevalence as 5% and the precision level as 5%, the minimum sample size was calculated as 73. Taking in consideration the non-response rate of 10%, a sample size of 80 was taken as final. Using two strata and considering that the major load of cases will be in the central hospitals than in provincial hospitals, 75% (total 60) of the data was collected from central hospitals and 25% of the data from the provincial hospitals (total 20). The cases were selected based on convenience. For qualitative part, exploratory sequential approach was done with in-depth interview taken from the selected participants. We planned to take maximum 25 interviews randomly from different institutes taking in account that 75% of the participant were from the central hospitals and 25% from the provincial hospitals. The numbers are based under assumptions that data saturation would take place at this point. However, we decided to stop with 15 interviews after a collective conclusion that all thematic heading has been repeatedly answered and it is less likely to lose some finding.

### Data collection and analysis

Data was collected by questionnaire method by a trained data enumerator who is well-versed and trained in CPR from among the hospital staff involved in performing CPR. Data was collected from the one of the participants involved in CPR as early as possible and not exceeding 10 days. The questionnaire was adapted as per the algorithms of the American Heart Association(AHA) [13]. Data was entered in the central entry system prepared by the investigating team. Data collection was monitored by the supervisor of the study. Interviewer are also advised to follow the cardiac arrest event every day at the hospitals and make efforts not to miss any data. In-depth interview was done purposively with audio recording for qualitative data after taking consent. The interviews were transcribed by an independent person trained in CPR from Nepali to English.

For data analysis, prevalence of CPR in different strata of the hospital was calculated taking into consideration all inpatients who were admitted in the emergency, hospital wards and intensive care units irrespective of duration of admission as the denominator. Other variables of study include outcome of arrest victim after CPR, occurrence of cardiac arrest at different sites of the hospital (emergency, intensive care unit, medical wards), training level of health care provider, estimated time from recognition to start of the CPR, conduct of chest compression and airway management, initial rhythm, use of monitor during CPR and outcome of arrest (S1 Table). Data enumerators were oriented before the collection of data. The principal investigator and the co-investigators supervised issues related to data collection. Qualitative variables were identified and expressed as numbers and percentages. Numerical variables were presented as mean and standard deviation. SPSS version 22 software was used to analyze the data. All the video interviews were recorded after consent and transcribed by independent person trained in CPR. The transcribed document was entered and thematic analysis was done using Dedoose 8.0.42. version. Thematic analysis was done as per AHA 2020 guideline IHCA chain of survival which is presented as follows (Table 1) [13].

**Table 1:** Different themes for cardiac arrest chain of survival as per American Heart Association 2020 guidelines [13].

S.N.	Themes	Categories
1.	Early recognition and prevention	Early recognition of arrest victim
		Surveillance system
2.	Activation of emergency response	Call for nearby help
		Call for resuscitation team
3.	High quality cardiopulmonary resuscitation	Early CPR
		Adequate rate
		Adequate depth
		Adequate recoil
		Minimum interruption
		Change of compressor
		Feedback
		Airway management
4.	Advanced resuscitative intervention	Recognition of rhythm
	(Defibrillation and Drugs)	Early defibrillation
		Drug use
		Treatment of reversible causes
5.	Post cardiac arrest care	Return of spontaneous circulation
		Infrastructure
		Multidisciplinary team
6.	Recovery	Rehabilitation

### Ethical approval and consent

Ethical approval was obtained from the Nepal Health Research Council (NHRC) (Ethical Review Committee Approval No:14612021P). Health personnel who had actively participated in the CPR were taken in-depth interviews using pre-formed questionnaire. For the quantitative part, written informed consent was taken before the interview, maintaining confidentiality. For the qualitative part, verbal consent was again taken before the start of the audio interview and recorded. Identity of the participant were not entered in the data sheet and any identifier that indirectly identified the participant were also deleted at the time of data entry. All this process was approved by the Ethical board of NHRC and the local institutional review board of the selected site.

## Results

Most of the arrest were 48.2 ± 17.1 (82–15) years of age with most interview of the healthcare provider conducted in 2.6 ± 2.4 (1–10) days (Table 2).

**Table 2:** Baseline characteristics of arrest victims and the healthcare provider.

Characteristics	Mean ± S.D
Age of the victim (years)	48.2 ± 17.1
Time from the cardiac arrest to interview of healthcare provider (days)	2.6 ± 2.4

The prevalence of the CPR in hospital was 1.92% with most higher incidences in the central hospitals 5.25% (Table 3).

**Table 3:** Prevalence of cardiopulmonary arrest at different hospitals*.

Hospitals	Number of inpatient admissions^#^(No. of cardiac arrest)	Prevalence* [95% Confidence Interval]
**Central hospitals**		
1. Hospital 1 (inside capital)	687 (40)	5.8% [0.04, 0.07]
2. Hospital 2 (outside capital)	424 (20)	4.7% [0.02, 0.07]
	1111(60)	5.4% [0.04,0.07]
**Provincial hospitals**		
1. Provincial hospital 1	1953 (10)	0.51% [0.002, 0.009]
2. Provincial hospital 2	1089 (10)	0.91% [0.004, 0.017]
	3042 (20)	0.65% [0.004, 0.010]
	4153 (80)	1.92% [0.01,0.02]

^#^ Includes all admission in emergency, wards, critical care units

* Calculated for 2 consecutive month study period

Most arrest happened in intensive care unit (Table 4) with only 26% [21] having the dedicated training on CPR (Table 5).

**Table 4:** Occurrence of cardiac arrest at different sites of the hospital.

S.N.	Sites	Number (%)	Prevalence* [95% Confidence Interval]
1.	Emergency	20 (25)	0.5% [0.003, 0.007]
2.	Inpatient ward	12 (15)	0.3% [0.001, 0.005]
3.	Intensive care unit	48 (60)	1.2% [0.009, 0.015]
	Total	80 (100)	

^#^Denominator (4153) includes all admission in emergency, wards, critical care units

* Calculated for 2 consecutive month study period

**Table 5:** Training level of the healthcare provider.

S.N.	Training level of healthcare provider	Number (%)
1.	**Training regarding cardiopulmonary resuscitation**	
	1. Short trainings a part of other training*	49 (61)
	2. Had dedicated training on cardiopulmonary resuscitation^#^	21 (26)
	3. Untrained	10 (12)
2.	**Training level of healthcare provider** ^**$**^	
	1. Medical officer	32 (40)
	2. Registrar^φ^	19 (23)
	3. Resident	7 (8.8)
	4. Nurse proficiency certificate level	16 (20)
	5. Nurse Bachelor level	2 (2.5)
	6. Health assistant	4 (5.0)
3.	**Duration when healthcare provided last attended cardiopulmonary resuscitation of any form**	
	1. In last 2 years	63 (78)
	2. 2–5 years	3 (3.8)
	3. Don’t remember	4 (5.0)
	4. Untrained	10 (12)

* Refers to training where cardiopulmonary was introduced as a part of the training, e.g., critical care training

^#^ Refers to dedicated training of cardiopulmonary resuscitation done by national and international societies, associations, universities, academy

^$^Refers to different educational degree provided as per the regulation of Government of Nepal

^φ^Refers to healthcare professional who have passed postgraduate training and is in first three years of service

Most of the CPR were recognized and started within 1.9 ± 1.4 (1–10) minutes and conducted for 23.5 ± 15.0 (2–80) minutes (Table 6).

**Table 6:** Time of recognition and total time of cardiopulmonary arrest.

**S.N.**	**Time duration** ^**#**^	**Mean ± S.D**
1.	Estimated time from recognition to start of CPR (minutes)	1.9 ± 1.4
2.	Estimated time from start of CPR to return of spontaneous circulation or exhaustion of the resuscitation team (minutes)	23.5 ± 15.0

^#^Time is based on arbitrarily from the respondent not by real calculation.

Asystole was the commonest rhythm observed. The second most frequent finding was difficulty interpreting rhythm. (Table 7).

**Table 7:** Initial rhythm, monitor use during CPR.

S.N.	Initial rhythm, monitor and defibrillator	Number (%)
1.	Initial rhythm	
	1. Asystole	53 (66)
	2. Difficult to interpret^#^	17 (21)
	3. Pulseless electrical activity (PEA)	4 (5.0)
	4. Pulseless ventricular tachycardia (VT)	4 (5.0)
	5. Ventricular fibrillation (VF)	2 (2.5)
2.	Use of monitor	
	1. Monitor with Electrocardiogram display	77 (96)
	2. Defibrillator monitor	1 (1.2)
	3. No monitor used	2 (2.4)

# Includes finding where there was difficulty interpretating rhythm in monitor, confusion in interpretation or had no monitor used.

Almost 11.25% (9) arrest have ROSC (Table 8) with most being transferred for post arrest care 10% (8).

**Table 8:** Outcome of arrest victim after revival.

S.N.	Status after revival (N = 33)	Number (%)
1.	Total case not revived	71 (88.8)
2.	Return of spontaneous circulation and transferred for post arrest care	9 (11.25)
	• Transferred to Intensive care unit	8 (10.0)
	• Referred to another center	1 (1.25)

Majority of the responses of the thematic analysis highlighted on the difficulties of early recognition and prevention, lack of clear-cut strategies for activation of emergency response system, poor performances and lack of feedback for chest compression, lack of provision for post cardiac arrest care and rehabilitation (Table 9).

**Table 9:** Responses for thematic analysis.

S.N.	Theme	Some of the important responses
**1.**	Early recognition and Prevention	*“The patient become unresponsive all of a sudden and carotid pulsation was not present*, *we started CPR immediately”* *“Since the patient’s neck region was swollen*, *there was difficulty in detecting carotid pulsation and blood pressure was not recordable when received*, *it was one of many difficulties to determine the need of CPR to the patient*.*”* *“We don’t have a system of getting prepared early for collapsing victim*, *the calls are usually taken by staffs on duty and then the on-call doctor are contacted*, *depending on the situation*. *We also call the specialist sometimes”*
**2.**	Activation of Emergency Response	*“I was the person on duty in the emergency*, *I called other staffs*, *we also called the doctor on duty”* *“We called the nursing staff nearby*, *called medical officer on duty*, *called anesthesiologist and started chest compression”* *“There was a nursing staff at the time*, *since the scenario was in the ICU*, *as a registrar*, *I was there along with the nursing staff*, *one medical officer and a medical officer from the Neuro department”*
**3.**	High quality Cardiopulmonary resuscitation	*“Due to lack of staff*, *there wasn’t a quality CPR*. *Chest compression and breathing were not performed as per the protocol”* *“While performing CPR*, *we were confused of the rate*, *depth*, *recoil”* *“We changed the compressor*, *but it wasn’t every two minutes”* *“We didn’t record it*. *We didn’t know whether it was 2 minutes*. *No*, *we didn’t change the compressor”* *“Due to lack of staff*, *there wasn’t anybody to provide feedback”* *“Firstly*, *we couldn’t find bag and mask and we had to search for it*. *Even after finding*, *we had difficulty performing bag and mask ventilation and there was confusion regarding endotracheal intubation*.*”*
**4.**	Advanced resuscitative intervention	*“There was no monitor in the wards*, *there was no ECG*, *no defibrillator*, *only saturation probe was possible while doing CPR”* *“The patient had multiple problems*, *so there might be many reasons*, *we didn’t know the exact cause*. *We couldn’t treat accordingly”*
**5.**	Post cardiac arrest care	*“It was case of MI*, *known case of extensive anteroseptal myocardial infarction*. *We couldn’t do thrombolysis from the onset for the duration of time*, *patient didn’t revive*.*”* *“After 3 cycles of CPR*, *patient had feeble carotid pulse*. *We couldn’t monitor properly since there was no monitor to observe*. *“* *“The patient was kept in mechanical ventilator*. *The pupils were dilated and fixed*. *He has many cycles of CPR*. *He was kept in mechanical ventilator for 1 hour*. *After that he went into asystole*.*”*
**6.**	Recovery	*“We have no provision for neurological rehabilitation in our hospitals*, *we generally take care as usual in intensive care unit after patient revival*.*”*

## Discussion

International Liaison Committee on Resuscitation (ILCOR) states that resuscitation in low resource setting must be calibrated and discussed to obtain consensus by experts from all settings [14]. Cardiac arrest observations are poorly practiced and reported in low-income countries and need to be improved before practice implementation [14]. Even tertiary care hospitals of low-income countries with intensive care services and emergency wards have a low rate of CPR performance, ROSC and 24-hour survival [6]. Lack of funding, ineffective CPR training, lack of infrastructure and resources, ineffective layman response has been cited as key factors for poor patient outcome in low- and middle-income countries [15]. It has been suggested that the usual protocol for CPR developed in middle and high income countries cannot be implemented real-time in low-income countries [15]. The African Federation for Emergency Medicine raises the issues of international guidelines not adequately addressing the local needs of low resource communities [16].

IHCA prevalence in our study is 1.92% with notable variation in central and provincial hospital with majority of events occurring in intensive care unit. A study done in Uganda in 2015 shows prevalence of 2.3% of IHCA with most cases occurring in critical care units and emergency and majority going unwitnessed [6]. In the United States, Medicare data for patients older than 65 documents the prevalence of cardiac arrest of 2.73 per 1000 population in hospital admissions from 1992–2005 [17]. There is evidence of regional variation from 2.33/1000 to 3.73/1000 hospital admission from the east side of the United States to the west which was partially explained by patient and hospital characteristics [18]. The high rate of prevalence in our context can be partly explained by rising burden of cardiovascular disease, lack of standard of care offered in high resource setting owing to a scarcity in financial, infrastructural and logistical resources and sufficiently qualified personnel [14, 19]. Almost 50% of the data in our study (Table 3) is from the trauma center, where there is likelihood of severe case admissions and high incidence of arrest, with peripheral hospital mostly catering all types of healthcare services which might possibly dilute the prevalence. Additionally, there is a tendency to refer serious cases to higher center which may partially explain the regional variation of prevalence in the study.

If we look at the rhythm at the time of arrest, the Indian online cardiac registry with 2121 cases shows asystole (67.7%) as the commonest rhythm, followed by PEA (25.6%), VT/VF (6.7%) [7]. In a study from Kenya, which is also a low-middle income country, 353 IHCA in different hospitals showed Non-shockable rhythms (Asystole: 47.6%, PEA: 38.2%, VT/VF: 5.4%, Unknown: 8.8%) for the majority of the cardiac arrests [20]. In our study, difficulty interpreting rhythm which is the second commonest finding (Table 7), may stem from multiple factors. These could include insufficient skills in rhythm recognition, malfunctioning or unavailable monitors, poor team dynamics and potential oversight by the interviewer due to multitasking during the event. It is also worth noting that majority of respondent in our study were untrained or partially trained, (Table 5) a finding in consistent with other study done in Nepal regarding CPR knowledge [21].

Our study shows 25% (20) of the patients had ROSC, out of whom 11.25% (9) patients were transferred for post-arrest care (Table 8). We did not have data on the 24-hour survival, and the number of patients discharged from the hospital. In Pakistan, a study done in the Emergency, out of 468 victims, 27.4% (128) had ROSC with 7.5% (35) survival to discharge.(26) In a small study done regarding IHCR in Uganda in 2015, 14 (7.4%) had ROSC, and only 3 (1.6%) had a 24-hour survival [6]. In another study done in 6 Kenyan hospital, ROSC occurred in 29.2% of patients and only 16 arrest (4.2%) were successfully discharged from the hospital [20]. In the Indian cardiac arrest registry, ROSC occurred in 355 (16.7%) patients, among whom 173 (8.2%) were alive and 14 (6.6%) has good neurological outcome [7]. In Thailand where facilities are well-equipped, it was interesting to see almost 62% cardiac arrest had a ROSC but survival to discharge was only 7% [22]. ROSC and patient improvement in neurological outcome is a collective effort of Chain of survival in AHA algorithm. In a Nigerian survey of 17 hospitals, which included most government hospitals that had the provision of intensive care services, it was found that only 20% hospitals had an appropriate cardiac arrest response team system, only 21% events were documented properly, and only 21% events were reviewed for educations and quality improvements. These are low-cost strategies for continuous quality improvements, which were ignored [23].

This study was done in a limited duration with efforts to include hospitals with the possibility of wide variations in service delivery. It is possible that interviewer might have missed some arrest event while following every day. Additionally, we stopped data collection upon reaching our desired sample which almost matched 2-month duration which might slightly underestimate prevalence. There are chances that interviewee might have missed important observations as he/she was busy doing one of the skills of the CPR. The annual hospital data, particularly of the peripheral hospital, might dilute CPR prevalence. Outside the hospital cardiac arrest which continued as IHCA and those occurring in the operation theatre were not recorded in our study which might underreport occurrences. A larger nationwide study done with hospitals of different tier’s delivering resuscitation services might give a clearer picture.

## Conclusions

IHCA is prevalent across many tiers of Nepal’s government healthcare system. The most common CPR rhythm is asystole, with approximately one tenth having ROSC and transferred for post arrest care. During various stages of chain of survival, there is inadequate recognition of cardiac arrest, poor performance and feedback during CPR and inadequate post-resuscitation care and rehabilitation. Additionally, there are insufficiently skilled healthcare personnel and a team dedicated to performing CPR. To improve the conduct and outcome of IHCR, a comprehensive system approach is recommended.

## Supporting information

S1 Table Questionnaire with consent. (DOCX)
