# Supplementary material for: Prevalence, outcome and conduct of in-hospital cardiopulmonary resuscitation in government hospitals of Nepal
Source: PLoS One. 2025 Jan 31;20(1):e0316950. doi: 10.1371/journal.pone.0316950 (PMC11785312; doi:10.1371/journal.pone.0316950)
Supplement: S1 Table — (DOCX) [file pone.0316950.s001.docx]

# Proforma

1. **INTRODUCTION**
2. Date and time of interview………………………….
3. Date and time of event…………………………………
4. Site of event (health facility name): ……………………
5. Interview taken from (any of the team members who has participated in Cardiopulmonary resuscitation)…………... ….
6. **INFORMATION CHECKLIST AND INFORMED CONSENT FORM**
7. **Information sheet for the participant**

Please find the details of the study for participant information as follows:

1. This is research to evaluate the prevalence, outcome and conduct of cardiopulmonary resuscitation in government hospital of Nepal.
2. This is mixed method study.
3. The participant is expected to read and brief the instructions and details of research and find a brief period of approximately 10 minutes to answer all the question in the questionnaire. This will also follow an audio interview which will be started after taking verbal consent.
4. The study helps to draw conclusion and recommendation regarding the prevalence, outcome and performance of cardiopulmonary resuscitation in government hospital of Nepal.
5. Participant will have full right to opt out of the study after reading the details of the study. The confidentiality of the participant will be ensured throughout the conduction of study.
6. This is a mixed method study and the benefits are indirect.
7. This study is done by group of experts working in government hospitals of Nepal. In case of any query related to research can be directed to the data collector or the principal investigator in following phone number (+9779841262246) and email address ([nawaph@gmail.com](mailto:nawaph@gmail.com)) .
8. The data can be shared for public benefit if required after investigator permission.
9. **Informed consent English**

**CONSENT FORM**

I give my consent for participating in the study “**Prevalence, outcome and conduct of in-hospital cardiopulmonary resuscitation in government hospitals of Nepal”** I have been fully communicated adequately about the details of the study before the start of the study and had full chance to deny my participation anytime during the conduction of the study. During the process, I have also been assured full confidentiality.

Signature of the respondent………………….

Date and time…………………………………

1. **Informed Consent Nepali**

सहमति फारम

म **“Prevalence, outcome and conduct of in-hospital cardiopulmonary resuscitation in government hospitals of Nepal”** भाग लिनका लागि आफ्नो सहमति दिन्छु। अनलाइन सर्वेक्षण सुरु हुनुभन्दा अघि मलाई अध्ययनको विवरणहरूको बारेमा पूर्ण रूपमा जानकारी गराइएको थियो र अध्ययनको सञ्चालनको बखत मेरो सहभागितालाई कुनै पनि समय अस्वीकार गर्ने पूर्ण अवसर पाएको छु। प्रक्रियाको क्रममा मलाई पूर्ण गोपनीयताको पनि आश्वासन दिइएको छ।

नाम: .....................................

ठेगाना:...................................

मिति र समय:....................................

1. **Quantitative Tool (Questionnaire)**
2. **RECOGNITION IN CPR, TRAINING LEVEL OF THE PROVIDER**
3. Estimated time from recognition to start of CPR (in minutes)……….
4. Place of CPR (Please encircle):
   1. Emergency
   2. Inpatient ward
   3. Intensive care unit
5. Age of the cardiac arrest victim (years): ……
6. Training level of the healthcare provider (Please encircle as appropriate):
   1. Medical officer
   2. Registrar
   3. Resident
   4. Nurse proficiency certificate level
   5. Nurse Bachelor level
   6. Health assistant
7. Training of CPR (Encircle as appropriate)
   1. Short trainings a part of other training
   2. Had dedicated training on cardiopulmonary resuscitation
   3. Untrained
8. Duration when healthcare provided last attended cardiopulmonary resuscitation training of any form (Encircle as appropriate)
9. In last 2 years
10. 2-5 years
11. Don’t remember
12. Untrained
13. **QUALITY OF CPR**
14. Use of monitor? (Please encircle)
    1. Usual monitor with ECG display
    2. Defibrillator monitor
    3. No monitor
15. Did they recognize the rhythm? (Yes/No) ........................................
16. What was the initial rhythm ? (Please encircle)
    1. Asystole
    2. Difficult to interpret
    3. Pulseless electrical activity
    4. Pulseless ventricular tachycardia
    5. Ventricular Fibrillation
17. Did the patient had return of spontaneous circulation (ROSC) (Answer Yes/No)?.....................
18. **POST CARDIAC CARE**
    - - 1. Estimate time from start of CPR to ROSC or exhaustion (in minutes)………….
        2. If revived, did he/she receive post cardiac care? (yes/no) ...................
        3. If yes what was done next (please encircle)
           1. Shifted to ICU for post cardiac care
           2. Referred to other hospital
19. **Qualitative Tool (In-depth interview guide):**

(This will be taken from audio interview after verbal consent.)

1. What is your experience on recognition of victim?

(Probing question on difficulties of recognition, skill issues)

1. How did the help arrive of arrest scene?

(Probing question on the system in the hospital, time of recognition and start of the CPR)

1. What was the quality of CPR? (Probing question of decision to start CPR, different qualities of CPR which includes rate, depth, recoil, change of compressor and feedback from team member)
2. How was the airway managed? (Probing question on bag and mask ventilation, confusion for intubation, timing with the chest compression)
3. Did you find any causes of arrest? (Probing question on reversible causes, treatment modalities and did it work?)
4. Did the patient revive? (Probing question on time of start of CPR to revival, how did you recognize return of spontaneous circulation, if no what was the cause of non-revival ?)
5. What was the status of the patient after revival? (Probing question on re-arrest, admitted in intensive care unit, referred to another center, how will be the patient be rehabilitated)
